# Environmental Governance Cooperative Behavior among Enterprises with Reputation Effect Based on Complex Networks Evolutionary Game Model

**DOI:** 10.3390/ijerph17051535

**Published:** 2020-02-27

**Authors:** Ming Luo, Ruguo Fan, Yingqing Zhang, Chaoping Zhu

**Affiliations:** 1 Economics and Management School, Guangxi Normal University, Guilin 541000, China; luoming831@163.com; 2 Economics and Management School, Wuhan University, Wuhan 430072, China; rgfan@whu.edu.cn (R.F.); chaopingzhu@126.com (C.Z.); 3 School of Management Science and engineering, Guizhou University of Finance and Economics, Guiyang 550025, China

**Keywords:** reputation effect, environmental governance, cooperative behavior, evolutionary game model in complex networks

## Abstract

This paper first portrays the equilibrium payoff of enterprise’s cooperation of environmental governance based on the Cournot model. Secondly, the evolutionary game model in complex networks is adopted to depict the evolution of environmental governance cooperative behavior among enterprises. Further, the evolutionary process of environmental governance cooperative behavior of enterprises is simulated considering the supervision behavior of government and the reputation evaluation behavior of environmental social organization. The results show that the cooperation level of enterprise group under self-organization condition will reach a low level; the supervision of government can enhance the cooperation level of enterprise group with high betrayal tempatation while it has limited effect on enterprise group with low betrayal tempatation. The reputation evaluation behavior of environmental social organization can realize reputation effect to improve the the cooperation level of enterprise group with high betrayal tempatation. The enhance of reputation sensitivity can optimize equilibrium distribution of reputation and it can strengthen the reputation effect on cooperation level. Based on the analysis above, the suggestions to effectively improve cooperation level are given.

## 1. Introduction

With the deepening of urbanization and industrialization in China, environmental pollution has become an important bottleneck restricting the high-quality economic development. As the main producer of environmental pollution, enterprises bear the important responsibility for environmental pollution governance. However, the diffusivity of environmental pollution leads to a significant positive externality of the environmental governance behavior of enterprises. As a result, some enterprises have the motivation to be *free riders*, that is, they do not pay the cost of environmental pollution governance, but they can share the benefit of pollution reduction [1]. Therefore, for environmental pollution governance, it is difficult to improve the governance performance only by relying on a single enterprise alone, and effective goal for environmental governance can be achieved through cooperation among enterprises [2]. If enterprises in a specific region are regarded as a group, the externalities of enterprises’ environmental governance behaviors will lead to *collective action dilemma*, which will affect the realization of cooperative governance of environmental pollution among enterprises.

The formation and evolution of environmental governance cooperative behavior among enterprises can be depicted as a dynamic evolutionary games process in which the agents of enterprise group finally reach an equilibrium state through mutual learning [3]. The evolutionary equilibrium state of environmental governance cooperative behavior among enterprises, on one hand, is related to tangible factors such as economic benefits and governance costs in the process of environmental pollution governance; on the other hand, corporate reputation, as an intangible asset, also has a significant impact on enterprise group cooperative behavior [4]. At the same time, in the developing process of current *multiple co-governance pattern* in China, the evolutionary driving force of corporate environmental governance cooperation is not only the endogenous driving force generated by the self-organization of enterprise group, but also the exogenous driving force generated by other subjects, such as government and environmental social organizations [5,6]. The environmental supervision behavior of government and the reputation evaluation behavior of environmental social organizations will have influence on the formation of enterprise cooperative behavior through making effect on economic benefits and corporate social reputation.

Therefore, this paper, from the perspective of multiple co-governance, introduce the reputation effect, applying the evolutionary game model in complex network to study the formation and evolution of enterprise group environmental governance cooperative behavior, so as to explore the evolutionary rules of environmental governance cooperative behavior among enterprises.

## 2. Literature Review

Viewed from existing literature, the researches on corporate environmental governance cooperative behavior are mainly carried out from the perspective of corporate production decision-making, because the environmental governance cooperation will directly affect corporate production cost and change the market structure, ultimately making an impact on corporate earnings. In current research, oligopoly competition model is an important tool to study the production cooperation decision-making among enterprises and how it lead to the change of market structure, and Cournot game model is the typical one to be focused by many scholars [7,8,9]. The cournot model is a game model with two firms in the market providing products, and there is no collusion between them, but each knows what the other will do, so as to determine the optimal output to maximize profits. Ferreira introduces residents’ environmental preference into the objective function of public enterprises and a mixed Cournot game involving environmental pollution control between domestic public enterprises and foreign private enterprises is analyzed [7]. Matsumoto et al. studied the government’s environmental charge collection behavior on specific or whole environmental pollution of enterprises and its influence on Cournot game among enterprises [8]. Xiao and Tian, based on Duopoly Cournot model, studied the selection of low-carbon environmental protection innovation mode for two types of enterprises, and simulated the dynamic evolution process of low-carbon innovation mode selection with evolutionary game model [9].

In the research on corporate environmental governance cooperation, the market relationships among enterprises is also an important factor which affects the corporate cooperative behavior. Relevant studies show that the relationships among enterprises in a group is essentially a complex network, whose topological structure can be characterized by complexity such as small-world and scale-free [10]. Therefore, the evolutionary game among enterprises can be regarded in essence as an evolutionary game issue in complex networks [11].

The first one to establish the research on evolutionary games in complex networks would be Nowak and May [12], and related researches kept emerging viewd from different network structures, including regular lattice [13,14,15], small-world networks [16,17], and scale-free networks [18,19,20]. As an effective tool, the evolutionary game in complex networks has good applicability for the research on group behavior of enterprises, and it is also applicable to study the environmental governance cooperation among enterprises. Wu et al. applied complex networks to construct the structure of enterprises in which the evolutionary process of the synergy game between government and enterprise is depicted [21]. Zhang and Fan combining the evolutionary game model in complex network and EWA learning algorithm, analyzes the emergence of low-carbon strategy competition in enterprise networks of industrial cluster, and carries out the scenario simulation [22]. The research above can indicate that the evolutionary games in complex networks can effectively depict the mechanism of mutual cooperation among enterprises.

In current multiple co-governance pattern, the evolutionary driving force of environmental governance cooperation among enterprises is not only including internal driving force formed by the interaction among agents of enterprise group, but also including the external driving force of government and environmental social organizations [23,24,25]. From the perspective of the government, its supervision behavior is essentially a punishment method to promote the formation of enterprises’ environmental governance cooperation, and punishment is an effective mechanism to promote cooperation level for evolutionary game in complex networks [26,27]. From the perspective of social organizations, they do not have the administrative power to punish enterprises directly, but they can shape the image of enterprises through public opinion transmission, which can be regarded as the reputation evaluation behavior of social organizations and reputation effect would be also an important factor to affect the cooperation level. In addition, other scholars made research and pointed out that reputation can, in various forms, improve the cooperation level in evolutionary game in complex network, but seldom scholars have applied this to the actual environmental governance issues [28,29,30,31].

Therefore, this paper will construct the model by introducing reputation effect and apply system simulation to explore the evolutionary process of cooperation among enterprises. The rest of the paper is structured as follows. The assumptions and the analytical framework of this paper are provided in Section 3 and an evolutionary game model of enterprise cooperative behavior is constructed in Section 4. Then the simulation algorithm is put forward in Section 5. Based on them, the simulation results are shown in Section 6 and the final section presents the conclusions and some policy implications.

## 3. The Assumptions and the Analytical Framework Under Complex Networks Context

### 3.1. Assumptions

In order to analyze the cooperative behavior among enterprises in environmental governance, several assumptions are provided as follow.

**Assumption** **1.** *We assume that the enterprise group consist of N enterprises, and there exists social network construction among the enterprises which is resulted at their social relationship. According to recent literature, the enterprise network can be depicted by small-word networks for its small-world characteristics. In this network, the node denotes the firm and, the edge e_ij_ shared by node i and node j denotes the product market where the products are provided by enterprise i and enterprise j.*

**Assumption** **2.** *The production of the firm will make pollution emission and it needs to carry on environmental governance. Suppose that the enterprises play two-player games between each two enterprises in network, and the strategy set of enterprise is {Comletely governance (C), Uncomletely governance (U)}. In the two-player game, if the players both select strategy C, we can say that the cooperative behavior is realized in this game. Therefore, we use the proportion of enterprises who selected strategy C to measure cooperation level of enterprises group during environmental governance.*

**Assumption** **3.** *Assumed that two enterprises belong to the same edge denotes that the two enterprises occupy this market and play Cournot game. The equilibrium of the game will determine the equilibrium price of the market, the equilibrium output of specific two enterprises and their equilibrium payoffs.*

**Assumption** **4.** *Government supervises the environmental governance behavior of enterprises. Meanwhile, environmental social organizations evaluate the reputation of enterprises and have an impact on the evolution of corporate reputation.*

### 3.2. The Framework Under Complex Networks Context

The relationships among enterprises always presents multiplicity, which means that an enterprise not only belongs to a single market, but also in multiple markets, and this forms the complex interaction among enterprises. If the enterprises are regarded as nodes and the market relationships are regarded as edges, the enterprise group can be depicted as a complex network with interlacing and multithreading structure.

In this paper, the reasons to use a complex network to depict the enterprise group structure can be explained from the following two aspects: (1) the analysis of the game among enterprises needs to rely on the network structure, because the network structure will have an impact on the final equilibrium, so it is closer to the reality taking the network structure of enterprise groups into consideration; (2) the evolution process of the game among enterprises involves strategy transformation, and their learning behavior is not only concerned with economic benefits, but also affected by reputation effect, while the spreading of reputation effect also needs the structure of group relationship as the action space. Therefore, it is more reasonable to explore the cooperation among enterprises in the analytical framework of complex networks.

**Figure 1:**
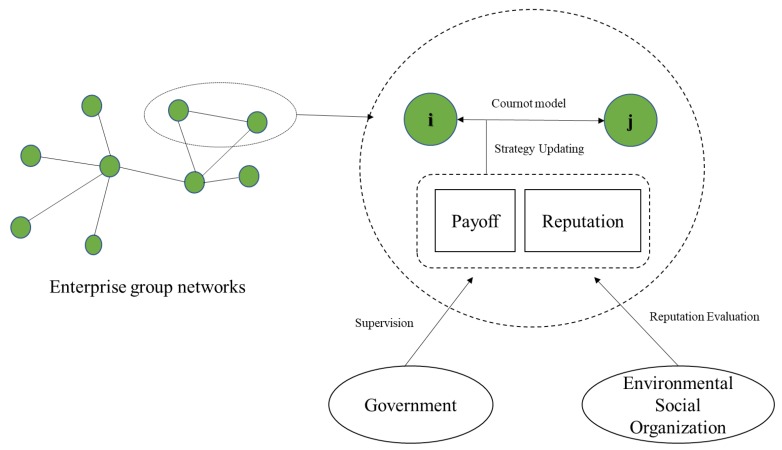
The enterprise group networks and the relationship among agents involved.

Under the complex network context, the analytical framework can be constructed as Figure 1. The enterprises in a group form a complex network. In these enterprises of this network, every two of them play a two-person game and the payoffs of the game are depended on the Cournot model. In the evolutionary process of enterprise cooperative behavior, the enterprises will update their strategies following certain rules and the updating rules will take the economic payoff, which is exogenously affected by government’s supervision, and the reputation, which is also exogenously affected by environmental social organizations’ reputation evaluation, into consideration. The simulation and exploration in this paper will be proceeded under this analytical framework.

## 4. The Evolutionary Game Model of Enterprise Cooperative Behavior

### 4.1. Cooperative Behavior Game Model Between Enterprises

Based on the assumptions above, the payoff matrix of the two-player game between enterprise *i* and enterprise *j* can be constructed as follow in Table 1:

And it is assumed that the payoff of enterprise *i* and enterprise *j* are depended by Cournot game model.

Suppose that the output of enterprise *i* is $q_{i}$ and the one of enterprise *j* is $q_{j}$. And a denotes the capacity of the market. Thenthe price of the market can be denoted by the inverse demand function:

(1) $$p=a-q_{i}-q_{j}$$

The production decisions of enterprises can be also influenced by environmental preference of consumers [32], so the the inverse demand function with environmental preference δ can be denoted as follow:

(2) $$p_{i}{}^{C}=a-q_{i}-\delta q_{j}$$

When δ = 1, it converts to be general inverse demand function. Then suppose that the marginal costs among enterprises are equal, namely $c_{i}{}^{C}=c_{i}{}^{U}=c$. And k is environmental pollution emissions per unit of output, $\lambda_{E}$ is the uncomplete extent of the enterprise when it selects strategy U, $e_{i}$ denotes the maximum quantity of pollution control is positively proportional to the actual quantity of pollution emission $kq_{i}$, that is $e_{i}=g_{\max}kq_{i}$, where $g_{\max}$ denotes the upper limit of an enterprise’s technical level in pollution control. In order to simplify, we set $\mu =g_{\max}k$, then $e_{i}=\mu q_{i}$. And we suppose that the pollution controlling cost of enterprises is quadratic function of quantity of pollution control, so the controlling cost of enterprise who selects strategy C or strategy U are respectively $\frac{1}{2}\gamma {\left(\mu q_{i}\right)}^{2}$ and $\frac{1}{2}\gamma {\left(\lambda_{E}\mu q_{i}\right)}^{2}$. According to the inverse demand function with environmental preference and the variables above, we can calculate the payoff of the enterprise, taking enterprise *i* for instance, under different strategy selections as follows:

(3) $$\pi_{i}{}^{U}=q_{i}p_{i}{}^{U}-q_{i}c-\frac{1}{2}\gamma {\left(\lambda_{E}\mu q_{i}\right)}^{2}$$

Based on the payoff functions constructed above, we can set three situations for different strategy selections between enterprise *i* and enterprise *j*: (1) enterprise *i* selects strategy C while enterprise *j* selects strategy *U*; (2) enterprise *i* and enterprise *j* both select strategy C; (3) enterprise *i* and enterprise *j* both select strategy *U*. In these three situations, we can obtain the equilibrium payoffs of enterprise *i* and enterprise *j* through solving the Cournot model between enterprise *i* and enterprise *j*, which contains the several steps: (1) constructing the payoff fuctions of enterprise *i* and enterprise *j* according to their strategy selections; (2) calculating their optimal reaction functions of the two enterprises by solving the first order condition; (3) obtaining the equilibrium output according to final equilibrium condition, and calculating the equilibrium payoff furtherly. After these steps, the equilibrium payoffs of the two enterprises in these three situations can be obtained as follows (The details to solve the Cournot model and obtain the equilibrium payoff are listed in Appendix A).

(4) $$\begin{array}{c} \Pi_{UC}=\pi_{j}=\frac{M_{1}(a-c)(\delta a-c)}{\left(\delta M_{1}+\delta M_{2}+M_{1}M_{2}\right)}-\frac{[2\delta M_{1}(M_{1}+M_{2})+(M_{2}-\delta -(1-\delta )a)M_{2}{}^{2}]{\left(a-c\right)}^{2}}{2{\left(\delta M_{1}+\delta M_{2}+M_{1}M_{2}\right)}^{2}}\\ \Pi_{CU}=\pi_{i}=\frac{M_{2}{\left(a-c\right)}^{2}}{\left(\delta M_{1}+\delta M_{2}+M_{1}M_{2}\right)}-\frac{[2\delta M_{2}(M_{1}+M_{2})+(M_{1}-\delta )M_{2}{}^{2}]{\left(a-c\right)}^{2}}{2{\left(\delta M_{1}+\delta M_{2}+M_{1}M_{2}\right)}^{2}}\\ \Pi_{CC}=\pi_{i}=\pi_{j}=\frac{\left(2+\gamma \mu^{2}\right)}{2{\left(\delta +2+\gamma \mu^{2}\right)}^{2}}{\left(a-c\right)}^{2}\\ \Pi_{UU}=\pi_{i}=\pi_{j}=\frac{\left(2+\gamma \lambda_{E}{}^{2}\mu^{2}\right)}{2{\left(3\delta +\gamma \lambda_{E}{}^{2}\mu^{2}\right)}^{2}}{\left(\delta a-c\right)}^{2} \end{array}$$

where $M_{1}=\delta +\gamma \mu^{2}$ and $M_{2}=(1-\delta )a+\delta +\gamma \lambda_{E}{}^{2}\mu^{2}$.

### 4.2. The Comparison of Enterprise Payoffs Under Different Situations

Based on the equilibrium payoffs from the Cournot model under three situations, the payoff matrix of the game can be constructed related to the corporate production decision. And we can find that if one of the game players selected the strategy U, the payoff of the two players will be affected by the uncomplete extent of pollution controlling $\lambda_{E}$. Define b = $1-\lambda_{E}$, through $\lambda_{E}$, the relationship among b and payoff in the game can be simulated in Figure 1 (The parameter setting is in keeping with the one in sub-Section 6.1).

**Figure 2:**
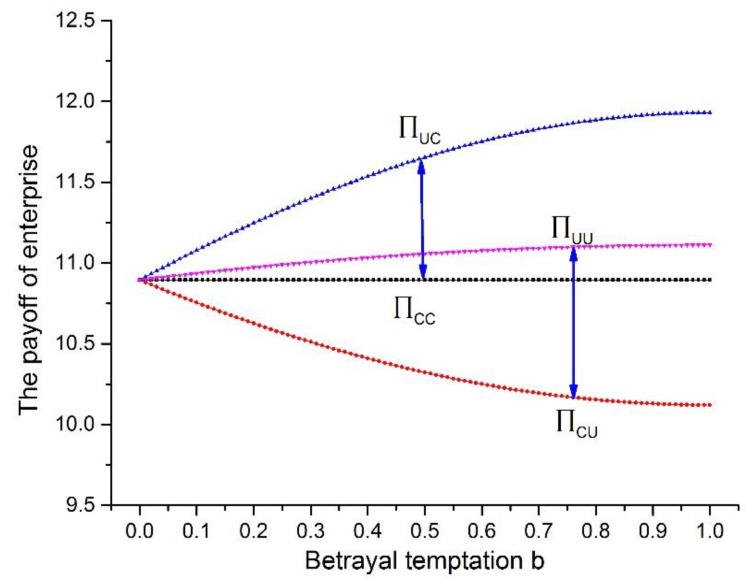
The payoff of enterprise under different strategy selection and betrayal temptation.

Suppose that the game players are enterprise *i* and enterprise j. From Figure 2, no matter which strategy the opponent enterprise *j* selects, the payoff of enterprise *i* obtains from selecting strategy C is always lower than the one from selecting strategy U, and the gap caused by strategy selecting will enlarge with the value of b increasing. The reason for this phenomenon could be that the enterprise selecting strategy C must pay more governance cost in pollution controlling and it also affects the production decision leading to the decrease of equilibrium payoff. The existence of payoff gap makes the enterprises tend to select strategy U. Therefore, the defined coefficient b can be set to represent the betrayal temptation, which manifests that the higher the value of b is, the more the payoff of enterprise selecting strategy U is. And it will lead to higher tendency that enterprise has to select strategy U and more serious cooperation dilemma in environmental governance.

## 5. Simulation Algorithm of Evolutionary Game

### 5.1. The Initialization of Enterprise Networks and Strategy Selection

According to relevant researches, the enterprise group presents a significant complex network structure and its complexity is reflected in the characteristics such as small-world, high aggregation and short network diameter, so the small-world networks can be applied to depict the structure of enterprise group [10]. This paper adopts the WS small-world networks, which is generated by algorithm provided by Watts and Strogatz [33]. The main idea contains the several steps as follow: (1) A regular network is generated with N nodes, whose average degree is Ks; (2)Based on the regular networks above, the rewiring probability pr is introduced, with which the edges can be broken and rewired. After the generation of small-world network, the strategies of nodes in networks need to be initialized. When *t* = 0, the proportion of nodes on the network selecting strategy C is 50% and the other 50% selecting strategy U, and the different strategies evenly distributed in networks.

### 5.2. Rules of the Game Among Enterprises

During the evolutionary process, every enterprise node in the networks will play two-player game with its neighborhood and obtain corresponding payoff in each evolutionary step. Defining the payoff obtained by enterprise *i* in the game with its neighbor enterprise *j* is $\Pi_{ij}(s_{i},s_{j})$, then the total payoff of enterprise *i* in this evolutionary step $\Pi_{i}$ can be denoted by the accumulated payoff that the enterprise *i* obtained in the games with its all neighbors:

(5) $$\Pi_{i}=\sum_{j\in \Omega_{i}}\Pi_{ij}(s_{i},s_{j})$$

where, $s_{i},s_{j}=C$ or U.

Government needs to supervise enterprises’ environmental governance practices. Suppose that the supervision intensity of government is r (0 < r < 1), which means that proportion of enterprises which are effectively supervised by government is r in every evolutionary step. The government levies environmental tax on enterprises in the process of environmental supervision and the environmental tax rate is supposed to be ϕ. When the government makes effective supervision on enterprise *i*:

(1) if the enterprise supervised has selected strategy C, the environmental tax should be calculated according to the direct pollution emission $kq_{i}-e_{i}$, and the payoff of this enterparise updates to be:

(6) $$\Pi_{i}^{S}=\Pi_{i}-\phi (kq_{i}-e_{i})$$

(2) if the enterprise supervised has selected strategy U, the environmental tax should be calculated according to the sum of direct pollution emission $kq_{i}-e_{i}$ and the quantity of the pollution which is uncompletely controlled $e_{i}-\lambda_{E}e_{i}$, then the payoff of this enterparise updates to be:

(7) $$\Pi_{i}^{S}=\Pi_{i}-\phi (kq_{i}-\lambda_{E}e_{i})$$

### 5.3. Strategy Updating Rules with Reputation Effect

After the games among enterprises and the supervision of government, enterprises have obtained payoff in this evolutionary step. Then the enterprises will make strategy updating by mutual learning. Accoring to relavent research, we can realize the strategy updating through Fermi rule, the probability that enterprise *i* will adopt the strategy of enterprise *j* is:

(8) $$\mathrm{Pr}(i\to j)=\frac{1}{1+\exp(\frac{\Pi_{j}^{S}-\Pi_{i}^{S}}{K})}$$

where *K* denotes the noise in strategy updating. And the tradional Fermi rule, like formular (28), only considers the economic payoff, while it ignores the reputation effect. In reality, the reputation effect is of importance in mutual learning among enterprises. This paper will introduce the reputation effect into mutual learning by the following two aspects:

(1) Define the reputation variable of enterprise *i* is $R_{i}$, and $R_{i}\in [1,R_{\max}]$. If the enterprise *i* adopts the strategy of enterprise *j* not only considering economic payoff, but also reputation. Then the payoff of players which takes reputation effect into consideration can be revised to be $\Pi_{i}^{SR}$, that is:

(9) $$\Pi_{i}^{SR}=\;{\left(\frac{R_{i}}{R_{\max}}\right)}^{\alpha }\Pi_{i}^{S}$$

where α denotes the reputation sensitivity of enterprises in mutual learning and α>0, which means that the higher the value of α is, the more the enterprises pays attention to. After introducing the payoff revised by reputation effect, the probability that enterprise *i* will adopt the strategy of enterprise *j* is also revised to be:

(10) $$\mathrm{Pr}(i\to j)=\frac{1}{1+\exp(\frac{\Pi_{j}^{SR}\;-\Pi_{i}^{SR}}{K})}$$

(2) Setting the updating rule of reputation $R_{i}$ to be:

(11) $$R_{i}(t+1)=R_{i}(t)+\Delta R_{i}(t)$$

The change of reputation $\Delta R_{i}(t)$ relies on the reputation evaluating behavior of environmental social organization (ESO). Suppose that the evaluating intensity of environmental social organization is u, which means that at the end of every step, proportion of enterprises will be evaluated by environmental social organization is u. The updating rule of $\Delta R_{i}(t)$ is as follow.

$$\Delta R_{i}(t)=\left\{ \begin{array}{l} -1\begin{array}{cc} & \mathrm{if}\;\mathrm{ESO}\;\mathrm{makes}\;\mathrm{evaluation}\;\mathrm{on}\;\mathrm{enterprise}\;\mathrm{and}\;\mathrm{enterprise}\;\mathrm{selects}\;\mathrm{strategy}\;U \end{array}\\ 1_{}{}_{}\begin{array}{cc} & \mathrm{if}\;\mathrm{ESO}\;\mathrm{makes}\;\mathrm{evaluation}\;\mathrm{on}\;\mathrm{enterprise}\;\mathrm{and}\;\mathrm{enterprise}\;\mathrm{selects}\;\mathrm{strategy}\;C \end{array}\\ 0_{}{}_{}\begin{array}{cc} & \mathrm{if}\;\mathrm{ESO}\;\mathrm{doesn}\text{’}t\;\mathrm{make}\;\mathrm{evaluation}\;\mathrm{on}\;\mathrm{enterprise} \end{array} \end{array}\right.$$

### 5.4. Circulation Rules

Based on the algorithm designed above, the whole evolutionary period is set to be TT, and the simulation of evolution process of cooperation among enterprises is conducted circularly on the basis of the steps above until the end of specified evolution period.

## 6. Simulation Results

### 6.1. The Parameters Setting

Without loss of generality, we set the quantity of enterprise group *N* = 1000, the average degree of the small-world networks Ks = 6, the rewiring probability to generate networks pr = 0.02. In the Cournot game between enterprises, a = 12, c = 2, γ = 1.1, μ = 0.3; the environmental tax rate *ϕ* = 1.5, and Rmax = 100; In the Fermi function for strategy updating of enterprises, the noise K = 0.1. Based on the parameter setting above, we can realize the simulation of the evolutionary process of environmental governance cooperative behavior through Matlab. During the simulating process, the evolutionary period TT = 1000, which denotes that a complete evolutionary process contains 1000 steps. The final simulating results are the average results obtained by 5 repeated simulation experiments, aiming to reduce the interference from system error.

### 6.2. The Effect of Environmental Preference δ

**Figure 3:**
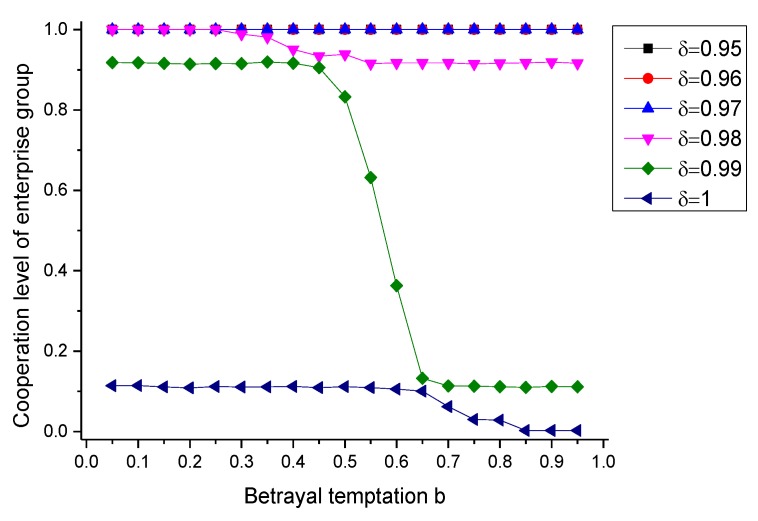
The evolutionary equilibrium under different environmental preference.

In the condition that government’s regulatory behavior and environmental social organization’s reputation evaluation behavior are absent, while there may exist environmental preference, we first conduct the simulation for the evolution of enterprises’ cooperative behavior, and the results are shown in Figure 3. When the environmental preference doesn’t work (δ = 1), the final equilibrium cooperation level of enterprise group is relatively low. If the betrayal temptation b is at a higer level (0.7 < b < 1), the equilibrium cooperation level is lower than 10%; As the betrayal temptation decreasing, the cooperation level slightly increases, and maintains at the level of 10% when 0 < b < 0.7. When environmental preference appears in the product market (the process that δ decreses from 1 to 0.95), the cooperation level can gradually increase to the complete cooperation level.

According to Figure 3, we can see that if there exists environment preference in product market, consumers are willing to bear higher price for the procuct provided by the enterprise who selects to make complete pollution controlling, and then through marketing adjustment mechanism, the enterprise can obtain indirectly a certain amount of compensation for the cost that it pays for environmental governance, which contributes to the existence of the environmental cooperative governance behavior. Therefore, if the environmental preferences can bring corresponding environmental dividends for the enterprise who selects cooperative behavior, it can benefit for the enterprise to tend to select cooperative strategy, and realize a higher cooperation level.

However, from the current situation of industrial development in China, the price gap between environment-protecting product and ordinary product is relatively small, for which the reasons can be summarized from two aspects. On one hand, the relatively slow industrial development in China leads to low environment-protecting added value of product, resulting that it is not conducive to the realization of price leverage of environmental protection product. On the other hand, the environment-protecting products in China are mainly positioned as middle-end or high-end products and the market proportion is small so that pollution-intensive products are still dominated by high-pollution industries, leading to the difficulty to promote the formation of environmental cooperative behavior among enterprises through marketing adjustment.

### 6.3. The Effect of Supervision Intensity r

**Figure 4:**
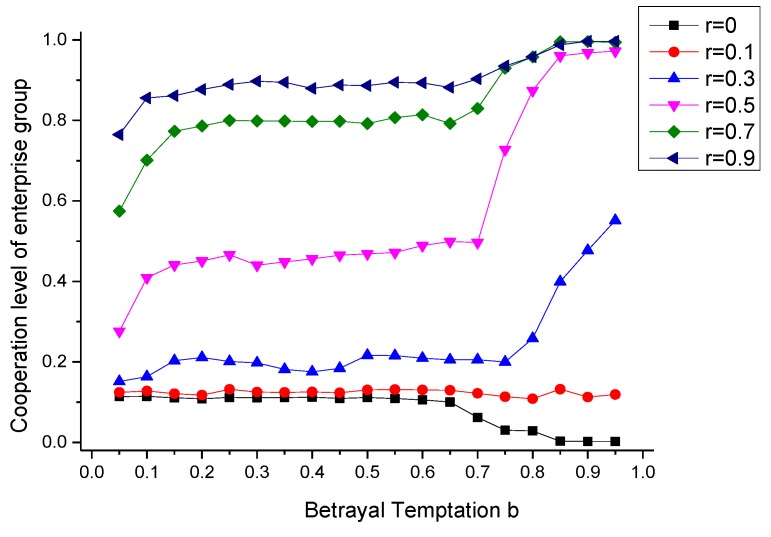
The evolutionary equilibrium under different supervision intensity.

For the fact that it is hard to realize environmental cooperative governance by marketing adjustment, the government’s supervision will be an approach to achieve the goal. Suppose that there doesn’t exist environmental preference (δ = 1) and the supervision intensity of government is r, levying the environmental tax for the pollution emitted by enterprises. The simulating results under different supervision intensity is shown in Figure 4.

According to Figure 4, the increase of government’s supervision intensity r can significantly improve the cooperation level of enterprise groups, but with the interval of b changing, the increasing extents of cooperation level from supervision intensity are different. This phenomenon denotes that the effect of government supervision behavior on enterprise groups cooperation is heterogeneous under different betrayal temptation conditions. When the betrayal temptation of environmental governance cooperation in an enterprise group is lower (0 < b < 0.7), although the government’s supervision intensity can inprove the cooperation level, the effect of supervision intensity is less effective than the condition under greater betrayal temptation (0.7 < b < 1). The main reason is that if the enterprise group faces a high level of betrayal temptation, the enterprises which select strategy U will bear a higher environmental tax under the effective supervision of government, demonstrating that the enterprises with speculative mentality will pay higher cost for its selection on strategy U under the effective supervision of government so that the enterprises with bounded rationality are forced to select the completely governance strategy (C) during the process of strategic updating, and achieve a higher level of cooperation level. However, if the enterprise group faces a low level of betrayal temptation, the enterprises that select uncompletely governance strategy (U) will under the effective supervision of government will be affected by a weak anti-driving effect from government’s environmental tax imposing, which makes it hard to promote the formation of cooperative behavior.

It can be seen from the analysis above that the government’s environmental supervision behavior can significantly promote the cooperation level of enterprise group, but the effect depends on the enterprise group’s betrayal temptation level and it shows significant *threshold effect*.

In actual industrial pollution control, the case with high betrayal temptation (0.7 < b < 1) mainly appears in energy-intensive industries, and under marketization condition, enterprises in energy-intensive industries tend to improve their output at the cost of environmental pollution because they will pay high opportunity cost if they participate in effective environmental governance cooperation. In this situation, through the leverage effect of environmental tax in supervision, the government can effectively internalize the pollution damage cost caused by these enterprises into their economic benefits, thus forcing these energy-intensive enterprises to participate in environmental governance cooperation and the cooperation level of can be improved effectively. However, enterprise groups with small betrayal temptation (0 < b < 0.7) always exist in high-tech industries with high environment-protecting level. However, enterprise groups with small betrayal temptation (0 < b < 0.7) always exist in high-tech industries with high environment-protecting level. Aiming at these enterprises, the government’s supervision behavior may not be able to achieve its expected effect, because that if the government’s supervision intensity fails to reach a certain degree, which means that the enterprise’s environmental tax imposed is not beyond its expected threshold-value range, making them bear the corresponding environmental tax rather than participate in cooperative behavior, or become temporizer in cooperation decision-making, further strenthing the speculation attitude of some enterprises to select strategy U, and making it hard to promote the cooperation level of enterprise group, resulting in governmental invalidity in environmental governance.

### 6.4. The Effect of Reputation Evaluation Intensity u

The government’s supervision behavior has limitations on the improvement of the cooperation level of enterprise groups with low-level betrayal temptation. In order to improve the cooperation level of enterprise group with low-level betrayal temptation, reputation effect is introduced into evolutionary analysis. And at the same time, the reputation evaluation behavior of environmental social organizations is also introduced, who play the role that it conducts third-party supervision on enterprise groups and evaluate their corporate reputation. In the case of considering the reputation effect, we study how the cooperative behavior is affected by the reputation evaluation intensity *u* of environmental social organizations.

**Figure 5:**
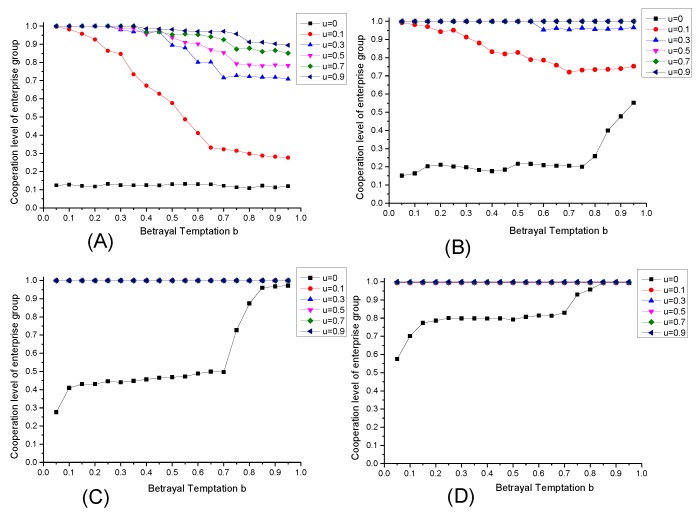
The evolutionary equilibrium under different reputation evaluation intensity. Note: α = 0.1, and (**A**) r = 0.1; (**B**) r = 0.3; (**C**) r = 0.5; (**D**) r = 0.7.

Firstly, we set that corporate reputation sensitivity α = 0.1, and set different supervision intensity r = 0.1, r = 0.3, r = 05, r = 0.7 respectively. Based on the setting above, the simulation is conducted and the results are shown in Figure 5.

According to Figure 5, it can be seen that the increase of reputation evaluation intensity can improve the cooperation level of enterprise group in Figure 5A–D. However, under different interval of b, we can also see that the increasing extents of cooperation level with increase of reputation evaluation intensity are different. This phenomenon denotes that environmental social organizations can play a significantly positive role in promoting the emergence of enterprise group’s environmental governance cooperation through the reputation effect in the evolution process on the basis of government environmental supervision, but the effects of reputation evaluation behavior of environmental social organization on enterprise groups are heterogeneous at different level of betrayal temptation. When the betrayal temptation of enterprise group’s cooperative behavior is low (0 < b < 0.7), the increase of reputation evaluation intensity will promote the cooperation level of enterprise group because the existence of reputation effect of environmental social organizations will weaken the superiority of enterprise selecting strategy U in strategy updating compared with the process that only the economic payoff is considered, which denotes that the reputation evaluation behavior of environmental social organization can make an effective control effect on speculation of the enterprises that select strategy U and government failure. And in the condition 0 < b < 0.7, the effect is more significant than the condition that betrayal temptation is larger (0.7 < b < 1). The reason is that in the situation 0 < b < 0.7, the temporizers in the evolution of enterprise group’s cooperation will observe that the gap of benefits under different strategy selections is small, and they may ignore the supervion behavior of government, making it difficult to achieve the anti-driving effect on cooperative behavior through supervision, while the reputation evaluation behavior of environmental social organizations can affect the evolution of the reputation, strengthening the reputation effect in mutual strategy learning, and guiding the bounded rational enterprises to attach importance to the formation of cooperation, so as to play a significant effect to promote the cooperation level of enterprise group. However, in the case of 0.7 < b < 1, the strategic learning of enterprises is more influenced by the government’s supervision, and the reputation effect in enterprises’ cooperation affected by environmental social organizations is relatively weaken. What’s more, the effect of reputation evaluation behavior of environmental social organizations is also affected by the supervision intensity of government. When the supervision intensity is low (r = 0.1 and r = 0.3), the reputation effect of environmental protection social organizations is weak, but when the supervision intensity increases (r = 0.5 and r = 0.7), the reputation effect of environmental social organization’s evaluation behavior is more significant.

### 6.5. The Effect of Corporate Reputation Sensitivity α

**Figure 6:**
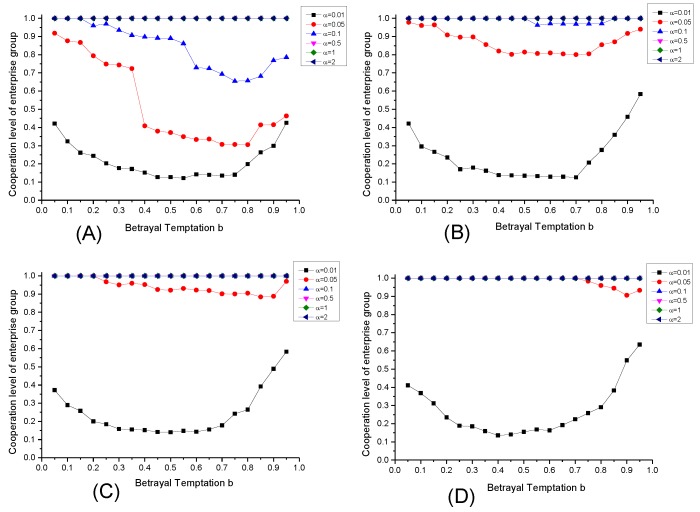
The evolutionary equilibrium under different corporate reputation sensitivity. Note: r = 0.3, and (**A**) u = 0.1; (**B**) u = 0.3; (**C**) u = 0.5; (**D**) u = 0.7.

In addition to the environmental social organization’s reputation evaluation intensity u, the corporate reputation sensitivity α will also have an impact on the evolution of cooperation level of enterprise group in the case that the reputation effect is taken into account. In order to study the influence of corporate reputation sensitivity α, we select r = 0.3 for typical case, and the case of u = 0.1, u = 0.3, u = 0.5 and u = 0.7 are set for simulation. The results are shown in Figure 6.

It can be seen from Figure 6 that under different level of corporate reputation sensitivity α, the enhancement of reputation evaluation intensity of environmental social organizations will produce different promoting effects on the cooperation level of enterprise group. When the corporate reputation sensitivity α is at a high level (α = 0.5 or α = 0.1), the cooperation level of enterprise group will significantly increase with the enhancement of reputation evaluation intensity of environmental social organizations. However, if the corporate reputation sensitivity α is at a relatively low level (α = 0.01), with the enhancement of u, the cooperation level of enterprise group does not change significantly, demonstrating that the reputation evaluation behavior of environmental social organization is difficult to promote cooperation level of enterprise group by guiding the strategy learning of enterprises under the condition that enterprise doesn’t attach importance to environmental governance reputation.

**Figure 7:**
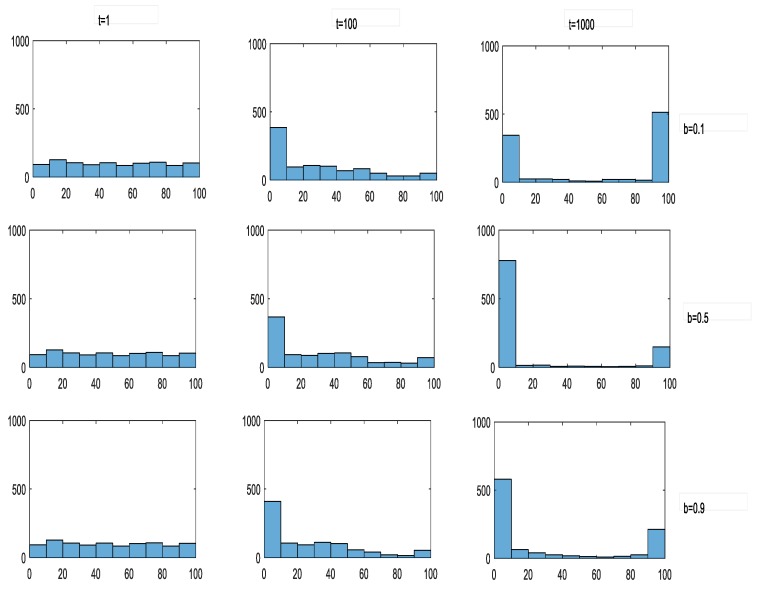
The evolution dynamic of reputation. Note: α = 0.01, r = 0.3, u = 0.3 and each row is b = 0.1, b = 0.5, b = 0.9 respectively.

It is worth noting that when α = 0.01, with the change of betrayal temptation, the change tendency of cooperation level of enterprise group presents a U-shaped curve. To explain this phenomenon, we can select the cases of b = 0.1, b = 0.5 and b = 0.9 to be examples. The reason for this phenomenon is that it is necessary in strategic learning of enterprises to comprehensively consider both the payoff gap and the reputation gap between the specific enterprise and its learning object. When the betrayal temptation is at a high level (b = 0.9), the payoff gap between enterprises is relatively larger, leanding to the polarization of final equilibrium distribution of corporate reputation in its evolution process (as Figure 7), demonstrating that the proportion of enterprises with high reputation is even with the one of enterprises with low reputation, so that there is a countervailing trend between the enterprises selecting strategy U (with high payoff and low reputation in equilibrium) and the enterprises selecting strategy C (with low payoff and high reputation in equilibrium), finally leading to the polarization of equilibrium strategy selection in the evolution of cooperation, the cooperation level maintaining at the level of 50%. As the betrayal temptation decrease to the situation of b = 0.5, the payoff gap caused by strategy selection also decreases. However, in the final equilibrium in this case, the reputation values of 80% of the enterprises will range from 0 to 10, while 20% will range from 90 to 100.Therefore, the narrowing of the payoff gap and the low distribution of reputation directly lead to the weakening of the countervalling trend, and the enterprises selecting strategy U dominate ultimately in the evolution, decreasing the cooperation level of enterprise group. As the temptation to betray continues to diminish to the situation of b = 0.1, the ultimate equilibrium reputation distribution switches back, namely that the proportion of enterprises with high reputation increases, and the cooperation level of enterprise group also enhances with it. Meanwhile, with the corporate reputation sensitivity α increasing, the equilibrium reputation distribution of enterprise group will beoptimized, and it can promote the cooperation level.

From analysis of the influence of corporate reputation sensitivity on evolution of enterprise group cooperation, in addition to government’s supervision behavior and reputation evaluation behavior of environmental social organization, it is also an important way to promote the level of corporate environmental governance cooperation to improve the environment-protecting awareness of enterprises and their attention to intangible assets such as environmental governance reputation.

## 7. Conclusions and Policy Implications

In this paper, the Cournot model is applied to construct payoff matrix of the game between enterprises, and then we adopt the evolutionary game in complex networks to construct the evolutionary game model of enterprise environmental governance cooperative behavior, and carries out the simulation analysis on its evolution process. With the detail analysis above, we can get some major conclusions as follows: (1) Under the self-organization evolution of enterprise group environmental governance, the cooperation level of environmental governance will eventually reach a low level;(2) The government’s environmental supervision behavior can effectively improve the cooperation level of enterprise group under high betrayal temptation, but supervision failure will appear in the enterprise group with low betrayal temptation;(3) The reputation evaluation behavior of environmental social organizations can make up for the limitation of government supervision through reputation effect and effectively improve the cooperation level of enterprise group with low betrayal temptation;(4) The low corporate reputation sensitivity is not conducive to the realization of the reputation effect through environmental social organization on the evolution of enterprise cooperative behavior and the enhancement of the reputation sensitivity of enterprises can optimize the equilibrium distribution of reputation and promote the cooperation level of enterprise group.

Based on the empirical analysis and major conclusions above, this paper puts forward some implications:

(1) For enterprises, they should be aware of that collective action dilemma of enterprise group in pollution controlling will not be conducive to the sustainable development of enterprise in the long run. To get out of this dilemma, the enterprise should not only pay attention to the present economic benefits, but also pay attention to the environmental governance reputation, improving the environment-protecting consciousness in the production and creating green reputation, in order to realize the exemplary role in the enterprise group.

(2) For the government, it is necessary to strengthen the supervision on enterprises’ environmental behavior and expand the scope of environmental supervision. In the process of supervision, grid management pattern in environmental supervision should be implemented, and the power and responsibility of relevant environmental supervision departments should be clearly allocated, so as to build a completed environmental supervision mechanism and achieve a situation in which environmental governance performance is effectively maintained.

(3) For environmental social organization, they should not only actively participate in third-party supervision, effectively restraining the environmental behavior of enterprises through the force of public opinion, but also positively carry out the environment-protecting education, improving enterprises’ emphasis on environmental governance behavior and environmental reputation. In this way anti-driving effect of reputation on cooperation of enterprise group can be effectively used to promote the sustainable development and realize win-win cooperation in environmental governance.

## Figures and Tables

**Table 1:** Payoff matrix of the game in environmental governance cooperation.

	Completely Governance (C)	Uncompletely Governance (U)
**Completely Governance (C)**	$\Pi_{CC}$, $\Pi_{CC}$	$\Pi_{CU}$, $\Pi_{UC}$
**Uncompletely Governance (U)**	$\Pi_{UC}$, $\Pi_{CU}$	$\Pi_{UU}$, $\Pi_{UU}$

## Appendix A

In Section 3.2, the details to calculate the equilibrium payoffs are shown as follows:

**Situation** **1.**
*If enterprise i selects strategy C and enterprise j selects strategy U, then*

(A1) $$\pi_{i}=q_{i}(a-q_{i}-\delta q_{j})-q_{i}c-\frac{1}{2}\gamma {\left(\mu q_{i}\right)}^{2}\;\pi_{j}=q_{j}\delta (a-q_{i}-q_{j})-q_{j}c-\frac{1}{2}\gamma {\left(\lambda_{E}\mu q_{i}\right)}^{2}$$

*Then the first order condition of two payoff functions are:*

(A2) $$\frac{\partial \pi_{i}}{\partial q_{i}}=a-c-2q_{i}-\delta q_{j}-\gamma \mu^{2}q_{i}=0\;\frac{\partial \pi_{j}}{\partial q_{j}}=\delta a-c-\delta q_{i}-2\delta q_{j}-\gamma \lambda_{E}{}^{2}\mu^{2}q_{j}=0$$

*Based on formula (A2), the optimal reaction functions of the enterprises can be obtained:*

(A3) $$q_{i}=\frac{a-c-\delta q_{j}}{2+\gamma \mu^{2}}\;q_{j}=\frac{\delta (a-q_{i})-c}{2\delta +\gamma \lambda_{E}{}^{2}\mu^{2}}$$

*Under the final equilibrium status,* $q_{i}$ *and* $q_{j}$ *will meet*

(A4) $$\frac{q_{i}}{q_{j}}=\frac{\left(\delta +\gamma \mu^{2}\right)}{[(1-\delta )a+\delta +\gamma \lambda_{E}{}^{2}\mu^{2})}$$

*Then the equilibrium output can be calculated as*

(A5) $$q_{i}=\frac{\left(a-c)[(1-\delta )a+\delta +\gamma \lambda_{E}{}^{2}\mu^{2}\right]}{\delta (\delta +\gamma \mu^{2})+(2\delta +\gamma \mu^{2})[(1-\delta )a+\delta +\gamma \lambda_{E}{}^{2}\mu^{2}]}\;q_{j}=\frac{\left(a-c)(\delta +\gamma \mu^{2}\right)}{\delta (\delta +\gamma \mu^{2})+(2\delta +\gamma \mu^{2})[(1-\delta )a+\delta +\gamma \lambda_{E}{}^{2}\mu^{2}]}$$

*To be simplified, we set* $M_{1}=\delta +\gamma \mu^{2}$ *and* $M_{2}=(1-\delta )a+\delta +\gamma \lambda_{E}{}^{2}\mu^{2}$ *and the equilibrium output can be rwritten as*

(A6) $$q_{i}=\frac{(a-c)M_{2}}{\delta M_{1}+\delta M_{2}+M_{1}M_{2}}\;q_{j}=\frac{(a-c)M_{1}}{\delta M_{1}+\delta M_{2}+M_{1}M_{2}}$$

*According to the equilibrium output, the equilibrium payoff of the two enterprises are*

(A7) $$\Pi_{CU}=\pi_{i}=\frac{M_{2}{\left(a-c\right)}^{2}}{\left(\delta M_{1}+\delta M_{2}+M_{1}M_{2}\right)}-\frac{[2\delta M_{2}(M_{1}+M_{2})+(M_{1}-\delta )M_{2}{}^{2}]{\left(a-c\right)}^{2}}{2{\left(\delta M_{1}+\delta M_{2}+M_{1}M_{2}\right)}^{2}}\;\Pi_{UC}=\pi_{j}=\frac{M_{1}(a-c)(\delta a-c)}{\left(\delta M_{1}+\delta M_{2}+M_{1}M_{2}\right)}-\frac{[2\delta M_{1}(M_{1}+M_{2})+(M_{2}-\delta -(1-\delta )a)M_{2}{}^{2}]{\left(a-c\right)}^{2}}{2{\left(\delta M_{1}+\delta M_{2}+M_{1}M_{2}\right)}^{2}}$$

*When* δ=1 *, namely the environmental preference doesn’t work, then the payoffs can be rewritten as*

(A8) $$\Pi_{CU}=\pi_{i}=\frac{{\left(1+\gamma \lambda_{E}{}^{2}\mu^{2}\right)}^{2}(2+\gamma \mu^{2})}{2{\left[2+\gamma \lambda_{E}{}^{2}\mu^{2}+\gamma \mu^{2}+(1+\gamma \lambda_{E}{}^{2}\mu^{2})(1+\gamma \lambda_{E}{}^{2}\mu^{2})\right]}^{2}}{\left(a-c\right)}^{2}\;\Pi_{UC}=\pi_{j}=\frac{{\left(1+\gamma \mu^{2}\right)}^{2}(2+\gamma \lambda_{E}{}^{2}\mu^{2})}{2{\left[2+\gamma \lambda_{E}{}^{2}\mu^{2}+\gamma \mu^{2}+(1+\gamma \lambda_{E}{}^{2}\mu^{2})(1+\gamma \lambda_{E}{}^{2}\mu^{2})\right]}^{2}}{\left(a-c\right)}^{2}$$

**Situation** **2.**
*If enterprise *i* and enterprise *j* both select strategy C, then*

(A9) $$\pi_{i}=q_{i}(a-q_{i}-\delta q_{j})-q_{i}c-\frac{1}{2}\gamma e_{i}{}^{2}\;\pi_{j}=q_{j}(a-\delta q_{i}-q_{j})-q_{j}c-\frac{1}{2}\gamma e_{j}{}^{2}$$

*Then the first order condition of two payoff functions are:*

(A10) $$\frac{\partial \pi_{i}}{\partial q_{i}}=a-c-2q_{i}-\delta q_{j}-\gamma \mu^{2}q_{i}=0\;\frac{\partial \pi_{j}}{\partial q_{j}}=a-c-\delta q_{i}-2q_{j}-\gamma \mu^{2}q_{j}=0$$

*The optimal reaction functions of the enterprises can be obtained:*

(A11) $$q_{i}=\frac{a-c-\delta q_{j}}{2+\gamma \mu^{2}}\;q_{j}=\frac{a-c-\delta q_{i}}{2+\gamma \mu^{2}}$$

*Under the final equilibrium status,*$q_{i}$ equals to $q_{j}$*, then*

(A12) $$q_{i}=q_{j}=\frac{a-c}{\delta +2+\gamma \mu^{2}}$$

*According to the equilibrium output, the equilibrium payoff of the two enterprises are*

(A13) $$\Pi_{CC}=\pi_{i}=\pi_{j}=\frac{\left(2+\gamma \mu^{2}\right)}{2{\left(\delta +2+\gamma \mu^{2}\right)}^{2}}{\left(a-c\right)}^{2}$$

*When* δ=1 *, the payoffs can be rewritten as*

(A14) $$\Pi_{CC}=\pi_{i}=\pi_{j}=\frac{\left(2+\gamma \mu^{2}\right)}{2{\left(3+\gamma \mu^{2}\right)}^{2}}{\left(a-c\right)}^{2}$$

**Situation** **3.**
*If enterprise *i* and enterprise *j* both select strategy U, then*

(A15) $$\pi_{i}=q_{i}\delta (a-q_{i}-q_{j})-q_{i}c_{i}-\frac{1}{2}\gamma {\left(\lambda_{E}e_{i}\right)}^{2}\;\pi_{j}=q_{j}\delta (a-q_{i}-q_{j})-q_{j}c-\frac{1}{2}\gamma {\left(\lambda_{E}e_{i}\right)}^{2}$$

*Then the first order condition of two payoff functions are:*

(A16) $$\frac{\partial \pi_{i}}{\partial q_{i}}=\delta a-c-2\delta q_{i}-\delta q_{j}-\gamma \mu^{2}q_{i}=0\;\frac{\partial \pi_{j}}{\partial q_{j}}=\delta a-c-\delta q_{i}-2\delta q_{j}-\gamma \mu^{2}q_{j}=0$$

*The optimal reaction functions of the enterprises can be obtained:*

(A17) $$q_{i}=\frac{\delta a-c-\delta q_{j}}{2\delta +\gamma \mu^{2}}\;q_{j}=\frac{\delta a-c-\delta q_{i}}{2\delta +\gamma \mu^{2}}$$

*Under the final equilibrium status,* $q_{i}$ *equals to* $q_{j}$ *, then*

(A18) $$q_{i}=q_{j}=\frac{\delta a-c}{3\delta +\gamma \lambda_{E}{}^{2}\mu^{2}}$$

*According to the equilibrium output, the equilibrium payoff of the two enterprises are*

(A19) $$\Pi_{UU}=\pi_{i}=\pi_{j}=\frac{\left(2+\gamma \lambda_{E}{}^{2}\mu^{2}\right)}{2{\left(3\delta +\gamma \lambda_{E}{}^{2}\mu^{2}\right)}^{2}}{\left(\delta a-c\right)}^{2}$$

*When* δ=1 *, the payoffs of the two enterprises are:*

(A20) $$\Pi_{UU}=\pi_{i}=\pi_{j}=\frac{\left(2+\gamma \lambda_{E}{}^{2}\mu^{2}\right)}{2{\left(3+\gamma \lambda_{E}{}^{2}\mu^{2}\right)}^{2}}{\left(a-c\right)}^{2}$$
